# IgA vasculitis in children

**DOI:** 10.1590/2175-8239-JBN-2022-E002

**Published:** 2022-02-11

**Authors:** Maria Goretti Moreira Guimarães Penido, Lilian Monteiro Pereira Palma

**Affiliations:** 1 Santa Casa de Belo Horizonte Hospital, Centro de Nefrologia, Unidade de Nefrologia Pediátrica, Belo Horizonte, MG, Brasil.; 2 Universidade Federal de Minas Gerais, Hospital de Clínicas, Faculdade de Medicina, Unidade de Nefrologia Pediátrica, Belo Horizonte, MG, Brasil.; 3 Universidade Estadual de Campinas, Hospital de Clínicas, Nefrologia Pediátrica, Campinas, SP, Brasil.

Immunoglobulin A (IgA) vasculitis (IgAV), classically known as Henoch-Schönlein purpura, is a type of non-thrombocytopenic small-vessel vasculitis and is the most frequent form of childhood systemic vasculitis (annual incidence: 3-26.7 per 100,000, depending on the country)^1^^-^ 3. IgAV is more frequent in childhood with a peak incidence around 4-6 years of age^2^^,^ 4. The disease usually presents with a palpable purpuric rash, gastrointestinal pain and bleeding, kidney involvement, arthralgia and/or arthritis^1^^-^ 4.Testicular inflammation (orchitis: 14% of male patients) is also seen, manifested by pain and swelling^5^.

IgAV has a seasonal variation, suggesting a role for environmental triggers and geographic distribution, and has a slight male predominance^5^. It occurs more frequently in certain parts of the world, such as Korea and Japan, but has equal distribution in all ethnicity grups^5^. The presentation in adults differs from that in children; adults rarely have abdominal pain and frequently have joint involvement^5^.

In 2013, the Revised Chapel Hill Consensus Conference defined IgAV at any age as a vasculitis with IgA-dominant immune deposits that affects small vessels, involving the skin, gut, and glomeruli, being associated with arthralgia or arthritis^6^. Kidney involvement is characterized by an IgA-dominant immune deposit indistinguishable from the pattern found in primary IgA nephropathy (IgAN). Criteria include a diagnosis of non-thrombocytopenic skin purpura with lower limb predominance as the main symptom and 1 of 4 additional criteria: 1) abdominal pain, 2) arthritis or joint pain, 3) kidney involvement (proteinuria >0.3 g/24h or a urine protein/creatinine ratio >30 mg/mmol on a spot morning sample or hematuria of >5 erythrocytes/high-power field), and 4) a leukocytoclastic vasculitis with predominant IgA deposits or kidney biopsy with predominant IgA deposits. Purpura with atypical distribution requires the presence of IgA deposits in the biopsy specimen^7^.

IgAV is considered a benign disease in children (around 94% of affected children achieve full and spontaneous recovery within two years), but its prognosis depends on the extent and the progression of kidney involvement^1^. Overall, 40-50% of affected children have kidney involvement ranging from microscopic hematuria to rapidly progressive glomerulonephritis, contributing to 1-2% of all chronic kidney disease (CKD) stage 5.^2^ In adults, the disease frequently occurs with atypical clinical features, severe kidney involvement, and with unfavorable outcome^3^. Recurrence (around 1/3 of patients) is more frequent in patients with kidney involvement and its duration is usually milder or shorter than the original episode^4^.

Genetics plays a role in determining the appearance of the disease and its severity, although there is no clear genetic correlation with primary IgAN^3^^,^ 5. It is very likely that the complement system is involved in addition to the coagulation and fibrinolytic systems^8^. Activation of the complement system in an environment of endothelial cell damage could lead to thrombotic microangiopathy^3^.

Currently, the hypothesis of an abnormal immune response to various antigens in genetically susceptible individuals is accepted^4^. It has been suggested that the first hit in the development of vasculitis lesions may be due to the formation of antibodies directed against the endothelial cells. These antibodies might be elicited by mimicry between microorganisms and endothelial cells with cross reactive production of IgA anti-endothelial cells^3^. Non-infectious agents such as medications, vaccines, and malignancies are also reported as triggering agents^4^.

The study by Kara *et al*., published in the current issue of Braz. J. Nephrol., used stringent criteria to select participants^9^. The diagnosis of IgAV was defined according to criteria accepted by the European Rheumatism Association (EULAR)^7^. Renal biopsies were classified according to the International Study of Kidney Disease in Children (ISKDC). Clinical outcome was graded according to modified Meadow's criteria^10^. Blood samples were assessed for hemoglobin, leukocytes and lymphocytes, neutrophil/lymphocyte ratio (NLR), platelets, mean-platelet volume, immunoglobulin A levels, and C-reactive protein. The frequency of gastrointestinal, scrotal, and kidney manifestations was documented. Steroids were used as first-line treatment and cyclophosphamide or cyclosporin-A were used in refractory cases.

Most patients were under 10 years of age, predominantly male, with skin and gastrointestinal involvement, and had hematuria and proteinuria at onset. Kidney biopsies were classified mostly as grade II and III according to ISKDC. Most patients were grade B (minor urinary abnormalities) and grade A (normal), respectively, on short- and long-term outcomes. The authors divided the patients in two groups (group I: grade I - II and group II: grade III - IV) according to ISKDC criteria and age group (≤10yr and >10yr). There was no difference between the groups (p=0.744).

The authors found significant correlation between scrotal involvement and unfavorable outcome in seven patients (14.8%). Only neutrophil/lymphocyte ratio was found significantly higher in those with scrotal involvement, which may be attributed to disease severity and inflammation^10^. According to Buscatti *et al.,* the scrotal involvement occurred in almost one fifth of IgAV patients, mostly as acute subtype^11^.

Kidney dysfunction, proteinuria, hypertension, and crescentic nephritis at onset were significantly associated to unfavorable outcome^3^. Shi *et al.* in a meta analysis showed that older age at onset, lower glomerular filtration rate, initial renal features of nephrotic syndrome and nephritic-nephrotic syndrome, and renal biopsy with crescentic nephritis were predictive of poor prognosis in children with IgAV^12^. Dyga and Szczepańska emphasize that most organ manifestations of IgAV are benign and self-limiting^4^. The study by Kara *et al*. report a significant correlation between hypertension and kidney impairment with unfavorable outcome^9^.

The recent European consensus (SHARE initiative) on diagnosis and treatment of IgAV provides a severity definition for IgAVN and recommends the prompt start of oral steroids (first-line) and IV pulses or immunosupression as second line treatment^13^. Mild IgAVN requires a more aggressive treatment with steroid pulses as first line treatment. ACE inhibitors are indicated as a supportive long-term therapy^3^^,^ 13. Akbalik Kara *et al.* treated all patients with steroids^9^. Fifteen were treated with oral cyclophosphamide for 8-12 weeks and complete remission was achieved within 3 to 6 months after beginning cyclophosphamide or cyclosporine-A. No significant difference was found in the effects of the two drugs on short- and long-term results.

The study by Akbalik Kara *et al*. contributes to the knowledge of IgAV and may change clinical practice. The three main messages are: (a) scrotal involvement in pediatric patients with IgAV can be associated with unfavorable outcome, (b) Cy-A and cyclophosphamide may be effective in steroid unresponsive IgAV pediatric patients, and (c) the disease has a good prognosis in children. However, other studies found that some children can progress to end-stage kidney disease. It seems reasonable that all IgAV pediatric patients, with no exception, should be periodically screened for urinalysis, blood pressure, and kidney function. Identifying children at greatest risk for progressive loss of kidney function is key to reducing the incidence of irreversible CKD. Hopefully, future studies will address these important questions.
